# The ongoing impact of Covid-19 pandemic on children with medical complexity: the experience of an Italian pediatric palliative care network

**DOI:** 10.1186/s13052-022-01206-9

**Published:** 2022-01-18

**Authors:** Veronica Grigoletto, Bianca Nardin, Valentina Taucar, Egidio Barbi, Lucia De Zen

**Affiliations:** 1 University of Trieste, Trieste, Italy; 2 Pediatric Palliative Care and Pain Service, Institute for Maternal and Child Health Burlo Garofolo, Trieste, Italy; 3 Pediatric Department, Institute for Maternal and Child Health Burlo Garofolo, Trieste, Italy

**Keywords:** Pediatric palliative care, Network, Health facilities, Home assistance, Sars-coV-2, Covid-19

## Abstract

**Background:**

Italy was the first European country to experience a massive outbreak of Sars-coV-2 in March 2020. Severe measures were introduced to face the pandemic, significantly impacting all healthcare services, including pediatric palliative care (PPC) networks. We investigated how the Covid-19 pandemic modified the provision of PPC services in Friuli Venezia Giulia, Italy. Both the acute and long-term impacts on the families were addressed.

**Methods:**

We administered a retrospective three-sections online questionnaire to the eligible families assisted by our regional PPC network. Inclusion criteria were: child needing specialistic PPC, adequate knowledge of the Italian language, being in charge of the PPC regional network of Friuli Venezia Giulia from February 1, 2020. The three sections examined the same issues in different periods: the pre-covid period (until February 29, 2020), the lockdown period (March 1, 2020, to April 30, 2020), and the post-lockdown period (May 2021).

**Results:**

Twelve patients were included. During the lockdown period, 54.6% of children had to stop physiotherapy sessions, while, among those who continued, 80.0% experienced a reduction in the sessions’ frequency. In the post-lockdown period, 45.5% of children did not have physiotherapy as often as before the pandemic onset. Overall, the access to medical visits during the lockdown and after its end was significantly reduced (*p* = 0.01).

The level of support perceived by the families descended from grade 3 (intermediate) in the pre-covid period to 2 (low) during the lockdown (*p* < 0.05) and returned to grade 3 in the post-lockdown period.

**Conclusion:**

The COVID-19 pandemic and the related restrictions impacted the families and caused a transitory contraction of the perceived support. The most significant change was reduced access to medical visits and physiotherapy, which lasted over a year after the start of the pandemic.

**Supplementary Information:**

The online version contains supplementary material available at 10.1186/s13052-022-01206-9.

## Background

The outbreak of the COVID-19 pandemic and the related control measures had a significant impact on hospital and territory health services in all countries [1]. Italy was the first European country to experience a massive outbreak [2] and introduced severe restrictions – including lockdown - in March 2020. These measures influenced several issues specific to palliative care, which is based on a person-centered multidisciplinary approach. Ordinary activities, such as providing a physical presence at patients’ homes, were severely limited to reduce the risk of contagion. Previous reports documented the effects of the pandemic period on adult palliative care networks, showing an increased pressure on patients, families and the workforce during the first months after the COVID-19 outbreak [3, 4]. The hospices and palliative care services staff faced considerable difficulties in providing adequate palliative care and support within the new pandemic context [5]. While few studies analyzed the effect of the acute phase of the pandemic on paediatric palliative care (PPC) services [6–9], little is known about the long-term consequences and the difficulties of the post-lockdown period for children and families assisted by PPC networks.

Our PPC regional network (Friuli Venezia Giulia, north-east area of Italy), instituted in September 2019, includes a reference center located in IRCCS Burlo Garofolo, Trieste, and all other actors involved in children’s assistance (local hospitals, territorial services, social co-workers, school, voluntary associations). The network is constantly growing and, up to October 2021, has been in charge of about 200 children, one-third of them with very specific and complex needs.

During all the pandemic period, including the strict lockdown decided by the Italian authorities for March–April 2020, the PPC staff continued to support parents by providing 24/7 telephone support and assistance, organizing home visits, and ensuring safe access to hospital services when necessary. The Italian authorities determined that routine activities - including check-ups and rehabilitation - were drastically reduced or suspended.

This study investigated how the COVID-19 pandemic modified the organization and provision of palliative healthcare services in our territory, addressing the acute and long-term impact on the families of children needing PPC.

## Methods

A retrospective three-sections online questionnaire was specifically developed and administered to the eligible families via e-mail by a medicine student between May 1 and 31, 2021. Families’ inclusion criteria were: having at least one child needing specialistic PPC (ACCAPED score > 50 [10]), being already in charge at the regional PPC network of Friuli Venezia Giulia from February 1, 2020, having adequate knowledge of the Italian language. The three sections examined the same issues in different periods, namely the pre-covid period (up to February 29, 2020), the lockdown period (March 1, 2020, to April 30, 2020), and the post-lockdown period, coincident with the time of the study (May 2021), more than one year after the beginning of the pandemic era. The questionnaire consisted of 85 statements and included a first section relating to patients’ age, residence, family, health needs, caregivers’ role, and economic situation. The core questions investigated the satisfaction of medical, social, and psychological needs in the different periods considered, including the well-being and the level of support perceived by the family. We developed the questionnaire starting from the FACETS-OF-PPC [11], a familiy-centered questionnaire which was recently validated for the comprehensive outcome measurement in children with severe neurological impairment needing PPC. The questionnaire’s structure was then adapted to allow a comparison between different time periods. Questions having nominal categorical variables as data output were structured as single answer multiple-choice questions, whereas questions involving ordinal categorical variables had the possible answers rated on a 5-point scale from 1 (not at all) to 5 (very much). A final open-ended question left the space to express the kind of assistance that the families most missed during and after the lockdown period. The estimated time to complete the questionnaire was about 15 min. The complete questionnaire is available as *Additional material*.

According to the research Institute policy, informed consent was signed by parents at the first visit. They agreed that “clinical data may be used for clinical research purposes, epidemiology, the study of pathologies and training, to improve knowledge, care, and prevention”. Furthermore, all families gave their informed consent to the study and agreed to data processing and publication.

### Statistical analysis

Descriptive analysis was performed by reporting frequencies and percentages for all the considered variables. Differences in the percentages of children with limited access to care between the three time periods were assessed with the Exact McNemar test, first comparing the pre-covid period with the lockdown period, then confronting the pre-covid period with the post-lockdown period (May 2021) to determine whether the situation had returned to normality. Wilcoxon test was used to evaluate variations in families’ well-being and perceived support. A *p*-value of less than 0.05 was considered statistically significant. All statistical analyses were performed using the Social Science Statistics website (www.socscistatistics.com).

## Results

We approached 20 eligible families, accounting for 22 children. Among these, 12 completed the questionnaire and agreed to participate in the study: 8 mothers, 2 fathers, and two custodial parents. Nine children (75.0%) were males, and the mean age was 8 years (range 1–16). All the patients included in the study had non-oncological chronic medical conditions. The main questionnaire results are shown in Table 1*.*

**Table 1:** Variations in the healthcare services provided in different periods

	Pre-lockdown	Lockdown	Post-lockdown	***p***-value
Number of patients	12	12	12	
Patients having physiotherapy, n (%)				
Yes	11 (91.7)	5 (41.7)	10 (83.3)	
The same as before the pandemic	–	1 (20.0)	6 (60.0)	0.002
Reduced	–	4 (80.0)	4 (40.0)	
No	1 (8.3)	7 (58.3)	2 (16.7)	
Patients attending medical checks, n (%)				
Yes	12 (100.0)	8 (66.7)	11 (91.7)	
The same as before the pandemic	–	5 (62.5)	7 (63.6)	0.001
Reduced	–	3 (37.5)	4 (36.4)	
No	–	4 (33.3)	1 (8.3)	
Patients having home nursing assistance, n (%)				
Yes	7 (58.3)	6 (50.0)	7 (58.3)	
The same as before the pandemic	–	4 (66.7)	5 (71.4)	0.125
Reduced	–	2 (33.3)	2 (28.6)	
No	5 (41.7)	6 (50.0)	5 (41.7)	

In most families (75.0%), both parents lived with the child; in 25.0% of cases, there was only one caregiver, always the mother. Most fathers (83.3%) had a steady job in the pre-covid period, while only one in four mothers (25.0%) had one. There were no variations in the employment rate during the considered periods.

In the pre-covid period, almost all children (91.7%) had access to physiotherapy, mainly at home (63.6%). During the lockdown period, less than half of the children (41.7% of all children) could continue their activity, whereas the majority had to stop physiotherapy sessions. Among those who continued during the lockdown, the great majority (80.0%) had to face a reduction in the sessions’ frequency. Looking at the post-lockdown situation, 9 children out of 11 (81.8%) have reobtained access to physiotherapy, about one-half (54.5%) with the same frequency of the pre-covid period. In our sample, the chance to have regular access to physiotherapy was significantly and permanently reduced after the outbreak of COVID-19 (*p* = 0.02).

**Fig. 1:**
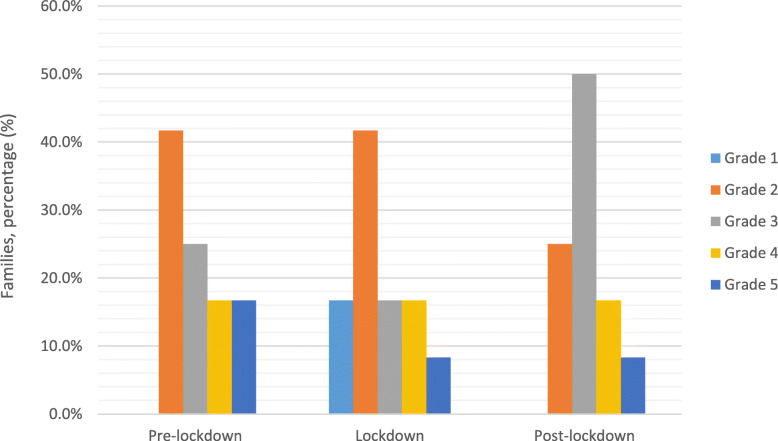
Variation in the perceived support in different time periods

Most children (58.3%) missed at least part of the planned medical checks during lockdown concerning medical visits. Some families (accounting for 33.3% of all children) stopped all medical visits fearing that they were infected with COVID-19. At the time of the study, most families (58.3%) reported accessing medical visits with the same frequency as before, while 33.3% still complained of a reduction, and one family did not attend medical visits at all to avoid a possible contagion. Overall, the access to medical visits during the lockdown and after it was significantly reduced (*p* = 0.01). Home nursing assistance in the pre-covid period was provided to more than half (58.3%) of the children in our sample. During the lockdown, most patients (85.7%) received support from nurses at home, but a third had to cope with a reduction in hours, which continued in all cases in the post-covid period. This variation did not reach statistical significance, but notably, the need for an increase in the hours of nursing assistance was the most frequent request (66.7%) in the open-ended question. During all periods, no differences emerged during or after the lockdown concerning family well-being, which was rated as grade 3 (on a scale of 1 to 5). Looking at the level of support perceived by the families, the great majority (83.3%) experienced some changes in the help they received during the lockdown, resulting in a lower grade of perceived support, which descended from grade 3 (intermediate) in the pre-covid period to 2 (low) during the lockdown (*p* < 0.05). Interestingly, the perceived support had returned to grade 3 at the time of the study, i.e., in the post-lockdown period (Fig. 1).

## Discussion

We conducted a retrospective analysis on patients in charge at our PPC network. For this reason, the sample size was small and our findings cannot be generalized. However, our results highlight some points that can be of general interst in the field of PPC. This study shiws that after the beginning of the Sars-coV-2 pandemic most families assisted by our PPC service experienced and perceived a significant decrease in the level of care. Families of children in need of palliative care have by definition a fragile balance, which was further challenged by several months of temporary limitations and social distancing, with intermittent school services and limited access to sport and care facilities [12]. As expected, during the lockdown in spring 2020, there was a significant reduction in the frequency of physiotherapy sessions and medical visits. This occurrence aligned with the limitation of non-urgent care the Italian Health Authorities decided to contain the virus spread. The possible consequences of this period of missing care have already been described in the literature and will only be quantifiable in the future [6]. However, this trend was not limited to the initial phase of the pandemic. Remarkably, more than one year after the pandemic emergency onset, several families still suffered from reduced access to these services. To a small extent, this may have been due to the family’s wish, but in most cases it depended on the territory and national health services. The reorganization of the public health system induced by the pandemic emergency led to wide waiting times for non-urgent care. Therefore, many routine specialistic visits were postponed. Furthermore, the PPC team in our territory did not include a dedicated physiotherapist since our patients have to rely on different territory services, depending on the area of residence. Targeted interventions were necessary to restore the pre-covid level of care and assistance, ensuring adequate and equal access to physiotherapy sessions and medical visits.

Contrary to previous reports [9], our data showed a reduction in perceived support. This occurrence was not unexpected, considering that these families suffered from a reduction of the provided services during a fearful and anxious time in a situation of social isolation. A 24/7 support was provided by the PPC team using extemporary telematic solutions, which may have been less effective than physical presence in the families’ perception. Consistently with this idea, the perceived support returned to the previous level after loosening the restrictions, i.e., when the palliative care team members had the chance to ensure regular home visits. In this context, the pandemic emergency showed that implementing telehealth services and making patients and caregivers familiar with them was essential to establish a modern end effective PPC network in our territory, as widely reported in the literature [13, 14].

Most families did not experience a significant reduction in nursing assistance; nevertheless, they used the final open-ended question to ask for an implementation of this service. This request represents a clear distress call, which we increasingly register in our daily activity, unable to address it providing concrete solutions. The healthcare services reorganization induced by the pandemic period led to a reallocation of most territory nurses, which were employed in hospitals or vaccine centers. Therefore, ensuring adequate in-home nursing assistance to PPC patients has become even more difficult in the post-lockdown period.

To our knowledge, this is the first report on the COVID-19 impact on PPC which includes data of the post-lockdown period. The study has several limitations, the main one being the small sample size, which is a consequence of the small number of patients assisted by our PPC network, recently established (in September 2019). The sample size was particularly limited because eligibility criteria included being already in charge of our PPC network in February 2020, only 5 months after the network creation. Due to the geographic position, near the national border, many families in our territory were not of Italian origin and did not speak fluent Italian; therefore, they could not be included in the project. Other limitations included the risk of recall bias and possible mistakes while completing the questionnaire.

## Conclusion

In conclusion, our findings proved that the COVID-19 pandemic and the related restrictions impacted the families assisted by our PPC network and caused a transitory decrease in the level of perceived support. The most significant change was reduced access to medical visits and physiotherapy, which continued more than one year after the onset of the pandemic.

A possible response to the needs that emerged from our study may come from a reorganization and enlargement of the PPC network with an increase in the number of dedicated health workers to provide adequate support to these families in this pandemic period and the future.

## Supplementary Information

**Additional file 1**. Questionnaire: the impact of Sars-coV-2 pandemic on the families of children with medical complexity.

## Data Availability

All data and materials relevant to this study are available from the corresponding author whenever required.

## Abbreviations

PPC: Pediatric palliative care
