# Supplementary material for: The ongoing impact of Covid-19 pandemic on children with medical complexity: the experience of an Italian pediatric palliative care network
Source: Ital J Pediatr. 2022 Jan 18;48:10. doi: 10.1186/s13052-022-01206-9 (PMC8764504; doi:10.1186/s13052-022-01206-9)
Supplement: Supplementary file 1 — Additional file 1. Questionnaire: the impact of Sars-coV-2 pandemic on the families of children with medical complexity. [file 13052_2022_1206_MOESM1_ESM.pdf]

Questionario

Benvenuta/o!
Vorrei innanzitutto ringraziarla per il suo tempo e la sua disponibilità.
Il questionario sarà articolato in 3 sezioni principali, più una breve sezione anagrafica iniziale:
-Analisi della situazione precedente al lockdown di marzo-aprile 2020
-Analisi della situazione durante il lockdown di marzo e aprile 2020
-Analisi della situazione attuale
La compilazione non dovrebbe richiedere più di 15 minuti, la ringrazio nuovamente e le auguro una buona compilazione!

---

*Campo obbligatorio

1. Acconsento a proseguire con il questionario consapevole che le mie risposte verranno trattate in forma anonima per le sole finalità di studio *

   Contrassegna solo un ovale.

   - Acconsento
   - Non acconsento

Anagrafica

2. Qual è il suo rapporto con il/la bambino/a afferente alla rete di Cure Palliative Pediatriche? *

   Contrassegna solo un ovale.

   - Padre
   - Madre
   - Genitore affidatario
   - Altro: ________________

3. Qual è il suo ruolo assistenziale? *

Contrassegna solo un ovale.

- Caregiver principale ed unico (non ci sono altri caregiver vicarianti)
- Assistenza condivisa (presenza di più caregiver equivalenti)

4. Dov'è la vostra residenza? (comune e provincia sono sufficienti) *

____

5. Qual è il vostro distretto di riferimento? *

____

6. Che grado di scuola frequenta suo/a figlio/a e dove è situata? (comune e provincia sono sufficienti) *

____

7. Per quanto riguarda la composizione del vostro nucleo familiare, i genitori sono conviventi? *

Contrassegna solo un ovale.

- Si
- No

8. Sono presenti fratelli o sorelle? *

Contrassegna solo un ovale.

- Si
- No

9. A quale fascia di reddito ISEE appartiene il vostro nucleo familiare? *

Contrassegna solo un ovale.

- Fascia 1 (da 0 a 23.120)
- Fascia 2 (da 23.121 a 27.000)
- Fascia 3 (da 27.001 a 31.000)
- Fascia 4 (da 31.001 a 40.000)
- Fascia 5 (40.001 a 50.000)
- Fascia 6 (da 50.001 a 63.000)
- Fascia 7 (da 63.001 a 75.000)
- Fascia 8 (da 75.001 a 95.000)
- Fascia 9 (oltre 95.001)
- Non so

10. Di quali ausili necessita suo/a figlio/a? *

Seleziona tutte le voci applicabili.

- [ ] Ausilii per la mobilizzazione
- [ ] Apparecchi per fisioterapia motoria
- [ ] Apparecchi per fisioterapia respiratoria
- [ ] Ventilatore meccanico
- [ ] Ossigenoterapia o alti flussi
- [ ] Monitor o saturimetro
- [ ] Nutripompa

Altro: [ ] ____________________

11. Quali devices sono presenti? *

Seleziona tutte le voci applicabili.

- [ ] Tracheostomia
- [ ] Gastrostomia
- [ ] Catetere venoso centrale o midline
- [ ] Catetere vescicale
- [ ] Digiunostomia

Altro: [ ] ____________________

Riabilitazione nel periodo PRIMA del lockdown

12. Suo figlio/a svolgeva riabilitazione con un fisioterapista prima del lockdown di marzo 2020? *

Contrassegna solo un ovale.

- ( ) Si
- ( ) No *Passa alla domanda 14.*

13. Dove? *

Contrassegna solo un ovale.

- ( ) A casa
- ( ) In distretto
- ( ) In altra struttura

Assistenza infermieristica PRIMA del lockdown

14. La sua famiglia riceveva assistenza infermieristica a domicilio? *

Contrassegna solo un ovale.

- ( ) Si
- ( ) No *Passa alla domanda 16.*

15. Per quante ore alla settimana circa? *

____

Supporto da parte di associazioni di volontariato PRIMA del lockdown

16. La sua famiglia ha ricevuto supporto da parte di associazioni di volontariato? *

Contrassegna solo un ovale.

- ( ) Si
- ( ) No *Passa alla domanda 18.*

17. Per cosa? *

Seleziona tutte le voci applicabili.

- [ ] Trasporto
- [ ] Accessi domiciliari
- Altro: [ ] ______________________

Scuola PRIMA del lockdown

18. Suo/a figlio/a frequentava la scuola? *

Contrassegna solo un ovale.

- ( ) Si
- ( ) No *Passa alla domanda 21.*

19. Dove? *

Contrassegna solo un ovale.

- ( ) A scuola
- ( ) A casa

20. Per quante ore a settimana? *

____

Supporto psicologico PRIMA del lockdown

21. La sua famiglia ha usufruito di supporto psicologico da parte di professionisti? *

Contrassegna solo un ovale.

- Si
- No *Passa alla domanda 23.*

Sezione senza titolo

22. Dove? *

Contrassegna solo un ovale.

- A casa
- In distretto
- In struttura

Moduli respiro PRIMA del lockdown

23. La sua famiglia ha potuto usufruire dei moduli respiro? *

Contrassegna solo un ovale.

- Si
- No *Passa alla domanda 25.*

Sezione senza titolo

24. Dove? *

Contrassegna solo un ovale.

- A casa
- In distretto
- Altro: ____________

Visite mediche PRIMA del lockdown

25. In che modalità avvenivano le visite mediche di suo/a figlio/a? *

Contrassegna solo un ovale.

- Con pediatra di libera scelta a casa
- Con pediatra di libera scelta in ambulatorio
- Con altri pediatri a casa
- Con altri pediatri in struttura
- Altro: ______________________

Attività lavorativa dei genitori PRIMA del lockdown

26. La madre lavorava? *

Contrassegna solo un ovale.

- Si
- No
- Non presente

27. E il padre lavorava? *

Contrassegna solo un ovale.

- Si
- No
- Non presente

28. Avevate la possibilità di avere qualche ora libera alla settimana? *

Contrassegna solo un ovale.

- Si
- No

Benessere e supporto PRIMA del lockdown

29. Per quanto riguarda il periodo precedente a marzo 2020 come valuta il livello di benessere complessivo (fisico, sociale, psicologico, spirituale...) di suo figlio/a in una scala da 1 a 5? Dove 1 indica un profondo stato di malessere, 3 una condizione neutra senza particolari difficoltà e 5 invece uno stato di completo benessere. (i valori 2 e 4 indicano situazioni intermedie rispettivamente tra 1 e 3 e tra 3 e 5) *

Contrassegna solo un ovale.

1	2	3	4	5
⬭	⬭	⬭	⬭	⬭

30. Per quanto riguarda il periodo precedente a marzo 2020 come valuta il livello di benessere complessivo (fisico, sociale, psicologico, spirituale...) della sua famiglia in una scala da 1 a 5?Dove 1 indica un profondo stato di malessere, 3 una condizione neutra senza particolari difficoltà e 5 invece uno stato di completo benessere. (i valori 2 e 4 indicano situazioni intermedie rispettivamente tra 1 e 3 e tra 3 e 5) *

Contrassegna solo un ovale.

1	2	3	4	5
⬭	⬭	⬭	⬭	⬭

31. Per quanto riguarda il periodo precedente a marzo 2020 come valuta il supporto ricevuto dalla sua famiglia in generale (dal sistema sanitario nazionale, dalle associazioni di volontariato, da familiari e amici...) in una scala da 1 a 5? Dove 1 significa assenza completa di supporto, 3 un supporto sufficiente e invece 5 il miglior supporto possibile per le esigenze della sua famiglia (2 e 4 indicano invece situazioni intermedie rispettivamente tra 1 e 3 e tra 3 e 5) *

Contrassegna solo un ovale.

	1	2	3	4	5
	⬭	⬭	⬭	⬭	⬭

Riabilitazione DURANTE il lockdown

32. Suo/a figlio/a ha svolto riabilitazione con un fisioterapista durante il lockdown di marzo-aprile 2020? *

Contrassegna solo un ovale.

- ⬭ Si, esattamente come prima *Passa alla domanda 34.*
- ⬭ Si, ma ridotta rispetto a prima
- ⬭ No *Passa alla domanda 34.*

Sezione senza titolo

33. A quanto si è ridotta? Per esempio se prima del lockdown si svolgeva 4 volte a settimana e durante il lockdown si è passati ad 1 sola, allora si è ridotta al 25% rispetto a prima *

Contrassegna solo un ovale.

- ⬭ 1-25%
- ⬭ 26-50%
- ⬭ 51-75%
- ⬭ 76-99%

Assistenza infermieristica a domicilio DURANTE il lockdown

34. Avete ricevuto assistenza infermieristica a domicilio durante il lockdown di marzo-aprile 2020? *

Contrassegna solo un ovale.

- Si, esattamente come prima *Passa alla domanda 36.*
- Si, ma ridotta rispetto a prima
- No *Passa alla domanda 36.*

Sezione senza titolo

35. A quanto si è ridotta? Per esempio se prima del lockdown avveniva 4 volte a settimana e durante invece solo 2 allora si è ridotta al 50% rispetto a prima *

Contrassegna solo un ovale.

- 1-25%
- 26-50%
- 51-75%
- 76-99%

Visite mediche DURANTE il lockdown

36. Le visite mediche necessarie a suo/a figlio/a si sono svolte durante il lockdown di marzo-aprile 2020? *

Contrassegna solo un ovale.

- Si, con la stessa frequenza di prima *Passa alla domanda 38.*
- Si, ma ridotte rispetto a prima
- No *Passa alla domanda 38.*

Sezione senza titolo

37. A quanto si sono ridotte? Per esempio se prima del lockdown avvenivano 2 volte a settimana e durante invece solo 1, allora si sono ridotte al 50% rispetto a prima

Contrassegna solo un ovale.

- 1-25%
- 26-50%
- 51-75%
- 76-99%

Accessi ospedalieri programmati DURANTE il lockdown

38. Gli accessi ospedalieri programmati (Es. Day hospital, ambulatorio) sono rimasti invariati durante il lockdown? *

Contrassegna solo un ovale.

- Si *Passa alla domanda 40.*
- No

Sezione senza titolo

39. Quanti accessi non sono stati effettuati? *

____

Supporto da parte di associazioni di volontariato DURANTE il lockdown

40. Avete ricevuto supporto da parte di associazioni di volontariato? *

Contrassegna solo un ovale.

- Si, esattamente come prima *Passa alla domanda 42.*
- Si, ma in forma ridotta rispetto a prima
- No *Passa alla domanda 42.*

Sezione senza titolo

41. A quanto si è ridotto? Per esempio se prima del lockdown il supporto veniva fornito circa 4 volte a settimana e durante il lockdown è sceso a 1 allora si è ridotto al 25% rispetto a prima *

Contrassegna solo un ovale.

- 1-25%
- 26-50%
- 51-75%
- 76-99%

Supporto psicologico DURANTE il lockdown

42. Avete ricevuto supporto psicologico? *

Contrassegna solo un ovale.

- Si, come prima *Passa alla domanda 44.*
- Si, ma in forma ridotta rispetto a prima
- No *Passa alla domanda 44.*

Sezione senza titolo

43. A quanto si è ridotto? Per esempio se prima del lockdown il supporto veniva fornito 4 volte a settimana e durante il lockdown è sceso a 2 allora si ridotto al 50% rispetto a prima *

Contrassegna solo un ovale.

- 1-25%
- 26-50%
- 51-75%
- 76-99%

Moduli respiro DURANTE il lockdown

44. Avete potuto usufruire dei moduli respiro? *

Contrassegna solo un ovale.

- ( ) Si, come prima *Passa alla domanda 46.*
- ( ) Si, ma in forma ridotta rispetto a prima
- ( ) No *Passa alla domanda 46.*

Sezione senza titolo

45. A quanto si sono ridotti? Per esempio se prima del lockdown ne usufruivate 4 volte al mese e durante invece solo 1 volta, allora si sono ridotti al 25% rispetto a prima *

Contrassegna solo un ovale.

- ( ) 1-25%
- ( ) 26-50%
- ( ) 51-75%
- ( ) 76-99%

Scuola DURANTE il lockdown

46. Suo/a figlio/a ha usufruito della Didattica a Distanza? *

Contrassegna solo un ovale.

- ( ) Si
- ( ) No *Passa alla domanda 48.*

Sezione senza titolo

47. In che modalità? *

Contrassegna solo un ovale.

- Con tutta la classe
- Con insegnante dedicato in forma 1 a 1
- Entrambi

Fornitura di farmaci e presidi DURANTE il lockdown

48. Avete potuto usufruire di una fornitura di farmaci e presidi? *

Contrassegna solo un ovale.

- Si, a casa
- Si, in distretto
- No

Attività lavorativa dei genitori DURANTE il lockdown

49. La madre lavorava? *

Contrassegna solo un ovale.

- Si, in presenza
- Si, in smart working
- No
- Non presente

50. Il padre lavorava? *

Contrassegna solo un ovale.

- Si, in presenza
- Si, in smart working
- No
- Non presente

51. Avevate la possibilità di qualche ora libera? *

Contrassegna solo un ovale.

- Si
- No

52. Avete riscontrato un cambiamento nella situazione economica della vostra famiglia? *

Contrassegna solo un ovale.

- No, è rimasta immutata
- Si, sono comparse delle difficoltà

Benessere e supporto DURANTE il lockdown

53. Per quanto concerne il periodo del lockdown, quindi marzo e aprile 2020, come valuta il livello di benessere complessivo (fisico, sociale. Psicologico, spirituale...) di suo figlio/a in una scala da 1 a 5?Dove 1 indica un profondo stato di malessere, 3 una condizione neutra senza particolari difficoltà e 5 invece uno stato di completo benessere *

Contrassegna solo un ovale.

	1	2	3	4	5	
Elevato malessere						Elevato benessere

54. Per quanto concerne il periodo del lockdown come valuta il livello di benessere complessivo (fisico, sociale, psicologico, spirituale...) della sua famiglia in una scala da 1 a 5?Dove 1 indica un profondo stato di malessere, 3 una condizione neutra senza particolari difficoltà e 5 invece uno stato di completo benessere. *

Contrassegna solo un ovale.

	1	2	3	4	5	
Elevato malessere	◯	◯	◯	◯	◯	Elevato benessere

55. Per quanto concerne il periodo del lockdown, come valuta il supporto ricevuto in generale dalla sua famiglia (dal sistema sanitario nazionale, dalle associazioni di volontariato, dai suoi familiari e amici...) in una scala da 1 a 5? Dove 1 significa assenza completa di supporto, 3 un supporto sufficiente e invece 5 il miglior supporto per le esigenze della sua famiglia. *

Contrassegna solo un ovale.

	1	2	3	4	5	
Assenza di supporto	◯	◯	◯	◯	◯	Massimo supporto

Riabilitazione nella situazione ATTUALE

56. Suo/a figlio/a sta svolgendo riabilitazione con un fisioterapista? *

Contrassegna solo un ovale.

◯ Si, come prima del lockdown *Passa alla domanda 58.*

◯ Si, ma in forma ridotta

◯ No *Passa alla domanda 58.*

Sezione senza titolo

57. A quanto si è ridotta? Per esempio se prima del lockdown si svolgeva 4 volte a settimana e adesso si è passati ad 1 sola, allora si è ridotta al 25% rispetto a prima *

Contrassegna solo un ovale.

- 1-25%
- 26-50%
- 51-75%
- 76-99%

Assistenza infermieristica a domicilio nella situazione ATTUALE

58. State ricevendo assistenza infermieristica a domicilio? *

Contrassegna solo un ovale.

- Si, come prima del lockdown *Passa alla domanda 60.*
- Si, ma in forma ridotta
- No *Passa alla domanda 60.*

Sezione senza titolo

59. A quanto si è ridotta? Per esempio se prima del lockdown si svolgeva 4 volte a settimana e adesso si è passati ad 1 sola, allora si è ridotta al 25% rispetto a prima *

Contrassegna solo un ovale.

- 1-25%
- 26-50%
- 51-75%
- 76-99%

Visite mediche nella situazione ATTUALE

60. La visite mediche necessario a suo/a figlio/a si stanno svolgendo? *

Contrassegna solo un ovale.

- Si, come prima del lockdown *Passa alla domanda 62.*
- Si, ma ridotte
- No *Passa alla domanda 62.*

Sezione senza titolo

61. A quanto si sono ridotte? Per esempio se prima del lockdown si svolgevano 4 volte al mese e adesso si è passati ad 1 sola, allora si è ridotta al 25% rispetto a prima *

Contrassegna solo un ovale.

- 1-25%
- 26-50%
- 51-75%
- 76-99%

Accessi ospedalieri programmati nella situazione ATTUALE

62. Gli accessi ospedalieri programmati (es. Day Hospital, ambulatori) per suo/a figlio/a si stanno svolgendo regolarmente? *

Contrassegna solo un ovale.

- Si *Passa alla domanda 64.*
- No, alcuni accessi non sono stati effettuati

Sezione senza titolo

63. Quanti accessi non sono stati effettuati? *

____

Supporto da parte di associazioni di volontariato nella situazione ATTUALE

64. State ricevendo supporto da parte di associazioni di volontariato? *

Contrassegna solo un ovale.

- Si, come prima del lockdown *Passa alla domanda 66.*
- Si, ma in forma ridotta
- No *Passa alla domanda 66.*

Sezione senza titolo

65. A quanto si è ridotto? Per esempio se prima del lockdown avveniva 4 volte al mese e adesso si è passati ad 1 sola, allora si è ridotta al 25% rispetto a prima *

Contrassegna solo un ovale.

- 1-25%
- 26-50%
- 51-75%
- 76-99%

Supporto psicologico nella situazione ATTUALE

66. State ricevendo supporto psicologico? *

Contrassegna solo un ovale.

- Si, come prima del lockdown *Passa alla domanda 68.*
- Si, ma in forma ridotta
- No *Passa alla domanda 68.*

Sezione senza titolo

67. A quanto si è ridotto? Per esempio se prima del lockdown avveniva 4 volte al mese e adesso si è passati ad 1 sola, allora si è ridotta al 25% rispetto a prima *

Contrassegna solo un ovale.

- 1-25%
- 26-50%
- 51-75%
- 76-99%

Moduli respiro nella situazione ATTUALE

68. Avete accesso ai moduli respiro? *

Contrassegna solo un ovale.

- Si, come prima del lockdown *Passa alla domanda 70.*
- Si, ma in forma ridotta
- No *Passa alla domanda 70.*

Sezione senza titolo

69. A quanto si è ridotto? Per esempio se prima del lockdown avveniva 4 volte al mese e adesso si è passati ad 1 sola, allora si è ridotta al 25% rispetto a prima *

Contrassegna solo un ovale.

- 1-25%
- 26-50%
- 51-75%
- 76-99%

Scuola nella situazione ATTUALE

70. Suo/a figlio/a frequenta la scuola? *

Contrassegna solo un ovale.

- Si
- No *Passa alla domanda 78.*

Sezione senza titolo

71. Per quante ore alla settimana? *

____

72. Frequenta scuola in presenza o in didattica a distanza? *

Contrassegna solo un ovale.

- In presenza *Passa alla domanda 73.*
- In didattica a distanza *Passa alla domanda 76.*

Scuola in presenza

73. Perchè ha scelto la modalità in presenza? *

Contrassegna solo un ovale.

- Difficoltà gestionali familiari (es. devo tornare a lavorare, non ho nessun parente che può stare a casa, non ho nessun aiuto territoriale)
- Benessere psicosociale di mio/a figlio/a
- Bisogno di mantenere un livello di istruzione adeguato ai pari di età
- Inadeguato progetto di istruzione domiciliare proposto
- Altro: _______________________________

74. Con che mezzo suo/a figlio/a si reca a scuola? *

Contrassegna solo un ovale.

- Autonomo
- Organizzato da terzi
- Mezzi pubblici
- Altro: ______________________

75. La scuola ha disposto delle protezioni particolari per suo figlio? *

Contrassegna solo un ovale.

- Si
- No

Passa alla domanda 78.

Scuola in DAD

76. Perchè ha scelto la modalità di didattica a distanza? *

Contrassegna solo un ovale.

- Paura del contagio vista la fragilità di mio/a figlio/a
- Possibilità di avere un adeguato progetto di istruzione domiciliare
- Possibilità di garantire assistenza a mio/a figlio/a durante la didattica a distanza
- Organizzazione familiare
- Altro: ______________________

77. Come si svolge la didattica a distanza? *

Contrassegna solo un ovale.

- Con tutta la classe
- Con insegnante dedicato in modalità 1 a 1
- Entrambi

Fornitura di farmaci e presidi nella situazione ATTUALE

78. State ricevendo forniture di farmaci e presidi? *

Contrassegna solo un ovale.

- Si, a casa
- Si, in distretto
- No

Attività lavorativa dei genitori nella situazione ATTUALE

79. In questo momento la madre lavora? *

Contrassegna solo un ovale.

- Si, in presenza
- Si, in smart working
- No
- Non presente

80. In questo momento il padre lavora? *

Contrassegna solo un ovale.

- Si, in presenza
- Si, in smart working
- No
- Non presente

81. Avete la possibilità di avere qualche ora libera? *

Contrassegna solo un ovale.

- Si
- No

Benessere e supporto nella situazione ATTUALE

82. In riferimento alla situazione attuale come valuta il livello di benessere complessivo (fisico, sociale, psicologico, spirituale...) di suo figlio/a in una scala da 1 a 5?Dove 1 indica un profondo stato di malessere, 3 una condizione neutra senza particolari difficoltà e 5 invece uno stato di completo benessere. *

Contrassegna solo un ovale.

	1	2	3	4	5	
Elevato malessere						Elevato benessere

83. In riferimento alla situazione attuale come valuta il livello di benessere complessivo (fisico, sociale, psicologico, spirituale...) della sua famiglia in una scala da 1 a 5? Dove 1 indica un profondo stato di malessere, 3 una condizione neutra senza particolari difficoltà e 5 invece uno stato di completo benessere. *

Contrassegna solo un ovale.

	1	2	3	4	5	
Elevato malessere	◯	◯	◯	◯	◯	Elevato benessere

84. In riferimento alla situazione attuale come valuta il supporto ricevuto in generale dalla sua famiglia (dal sistema sanitario nazionale, dalle associazioni di volontariato, dai suoi familiari e amici...) in una scala da 1 a 5? Dove 1 significa assenza completa di supporto, 3 un supporto sufficiente e invece 5 il miglior supporto per le esigenze della sua famiglia. *

Contrassegna solo un ovale.

	1	2	3	4	5	
Assenza di supporto	◯	◯	◯	◯	◯	Massimo supporto

85. Questo spazio è a Sua disposizione per ulteriori suggerimenti in merito al possibile miglioramento della gestione dell'assistenza a sua/o figlia/o e alla sua famiglia

Questi contenuti non sono creati né avallati da Google.

Google Moduli
